# High throughput RNA sequencing of a hybrid maize and its parents shows different mechanisms responsive to nitrogen limitation

**DOI:** 10.1186/1471-2164-15-77

**Published:** 2014-01-28

**Authors:** Yong-Mei Bi, Ann Meyer, Gregory S Downs, Xuejiang Shi, Ashraf El-kereamy, Lewis Lukens, Steven J Rothstein

**Affiliations:** 1 Department of Molecular and Cellular Biology, University of Guelph, N1G 2 W1 Guelph, ON, Canada; 2 Department of Plant Agriculture, University of Guelph, N1G 2 W1 Guelph, ON, Canada; 3 Clinical Genomics Centre, The UHN/MSH Gene Profiling Facility, M5G 1X5 Toronto, ON, Canada; 4 Current address: Horticulture Department, Faculty of Agriculture, Ain Shams University, 11241 Cairo, Egypt

**Keywords:** Maize, mRNA-Seq, Nitrogen limitation, Nitrogen use efficiency, Hybrid, Inbred, Allelic expression

## Abstract

**Background:**

Development of crop varieties with high nitrogen use efficiency (NUE) is crucial for minimizing N loss, reducing environmental pollution and decreasing input cost. Maize is one of the most important crops cultivated worldwide and its productivity is closely linked to the amount of fertilizer used. A survey of the transcriptomes of shoot and root tissues of a maize hybrid line and its two parental inbred lines grown under sufficient and limiting N conditions by mRNA-Seq has been conducted to have a better understanding of how different maize genotypes respond to N limitation.

**Results:**

A different set of genes were found to be N-responsive in the three genotypes. Many biological processes important for N metabolism such as the cellular nitrogen compound metabolic process and the cellular amino acid metabolic process were enriched in the N-responsive gene list from the hybrid shoots but not from the parental lines’ shoots. Coupled to this, sugar, carbohydrate, monosaccharide, glucose, and sorbitol transport pathways were all up-regulated in the hybrid, but not in the parents under N limitation. Expression patterns also differed between shoots and roots, such as the up-regulation of the cytokinin degradation pathway in the shoots of the hybrid and down-regulation of that pathway in the roots. The change of gene expression under N limitation in the hybrid resembled the parent with the higher NUE trait. The transcript abundances of alleles derived from each parent were estimated using polymorphic sites in mapped reads in the hybrid. While there were allele abundance differences, there was no correlation between these and the expression differences seen between the hybrid and the two parents.

**Conclusions:**

Gene expression in two parental inbreds and the corresponding hybrid line in response to N limitation was surveyed using the mRNA-Seq technology. The data showed that the three genotypes respond very differently to N-limiting conditions, and the hybrid clearly has a unique expression pattern compared to its parents. Our results expand our current understanding of N responses and will help move us forward towards effective strategies to improve NUE and enhance crop production.

## Background

Nitrogen (N) is the most important inorganic nutrient for plant growth. The production of high-yielding crops is associated with the application of large quantities of N fertilizers [1]. The addition of N fertilizer is typically the single highest input cost for many crops and since its production is energy intensive, this cost is dependent on the price of energy [2]. Incorporation of N into agricultural crops, however, rarely exceeds 40% of the applied N, indicating a serious inefficiency in N utilization [3,4]. The remaining N from fertilizer is lost to the atmosphere or leached to the groundwater and other freshwater bodies, which is causing serious N pollution and becoming a threat to global ecosystems [3,4]. Therefore, to minimize the loss of N, reduce environmental pollution and decrease input cost, it is crucial to develop crop varieties with high nitrogen use efficiency (NUE) [5,6].

While improved agricultural practice is one way to increase NUE [7], it is also crucial to understand more about the genetics of NUE in order to select better varieties. Several studies have presented evidence that natural variation exists in Arabidopsis for nitrogen metabolism, including nitrogen uptake and nitrogen remobilization (reviewed by [8-10]). Genetic differences in N uptake and/or grain yield per unit of N applied have also been reported in different crops including wheat, rice, maize, sorghum, and barley [11-16].

Maize is one of the most important crops cultivated worldwide and a large amount of fertilizer is used for its production. Genetic variation in maize such as in N-remobilization and post-silking N-uptake, nitrogen metabolism, nitrogen management, and senescence have been reported [17-21]. Although some physiological and phenotypic analyses have been done [1], the molecular knowledge governing genetic variation among different varieties for NUE is poorly understood. In a previous study, we developed a hydroponic growth system and tested two inbred lines and their hybrid that were different in their NUE at maturity under N limitation [22]. One parent, SRG200, showed a higher NUE than the other parent SRG100. Differences between these genetic lines were found after phenotypic, molecular, and metabolic factors were tested at an early vegetative stage and transcriptional analysis on a small number of selected genes involved in N metabolism was conducted [22]. To have a better understanding of how different maize genotypes respond to N limitation, we used whole transcriptome sequencing (mRNA-Seq) to conduct a survey of the transcriptomes of these SRG100, SRG200, and their hybrid under sufficient and limiting N conditions. The primary objectives of this study were to observe the major differences in gene expression among these three lines responding to N limitation, to distinguish the contribution of each parental line to gene expression in the hybrid line, and to discover whether these differences in expression correlate with the differences in the NUE trait studied.

## Results

### Transcriptomes of the two inbred parental lines and the hybrid line under sufficient and limiting N conditions

The two inbred lines, SRG100 and SRG200, and the hybrid line, SRG150, were grown under sufficient (3 mM) and limiting (1 mM) N conditions as described previously [22]. To profile the transcriptome, mRNA from leaves and roots of SRG100, SRG200, and SRG150 plants grown under the two N conditions were extracted, fragmented, and used for cDNA synthesis. Libraries were constructed and mRNA-Seq was performed (see details in Methods). 64-79% of total paired reads were aligned to the B73 reference genome (Table 1) and aligned sequences were assembled with Cufflinks guided by a reference annotation from Ensembl Genomes (zea_mays.AGPv2.62.gtf). The completed assembly contains 119,020 genes (the maize working gene set (WGS), the set of evidence-based and predicted genes, has 110,028 genes, and the filtered gene set (FGS), a subset of the WGS, has 39,656 genes). The percentage of mapped reads, genes with fragments per kilobase of exon per million mapped reads (FPKM) > 0, > 1, or >  5 are summarized in Table 1. A transcript was considered to be expressed if its normalized expression value was greater than one FPKM [23,24], and if it was part of the FGS version 5b.60 (maizesequence.org). More genes were expressed under N limitation than under sufficient N, with a 4-8% higher number if the FPKM cutoff is greater than 1, and with a 9-26% higher number if FPKM cutoff is greater than 5 (Table 1). The original datasets have been deposited in the Sequence Read Archive (SRA), with the accession ID SRP033653 and the following link: http://www.ncbi.nlm.nih.gov/sra/?term=SRP033653.

**Table 1:** Expressed genes in all the samples

**Genotype**	**N condition**	**Tissue**	**% of total paired reads**	**# genes (FGS) >0 FPKM (% difference under N limitation)**	**# genes (FGS) >1 FPKM (% difference under N limitation)**	**# genes (FGS) >5 FPKM (% difference under N limitation)**
SRG100	Limiting	Leaf	75.97	28048 (−3.2)	18783 (5.9)	11543 (15)
SRG200	Limiting	Leaf	77.57	26022 (−7.5)	17953 (8.0)	10496 (20)
SRG150	Limiting	Leaf	77.35	27578 (−4.6)	18506 (7.5)	10785 (20)
SRG100	Limiting	Root	75.49	29527 (−2.8)	21314 (8.3)	13574 (26)
SRG200	Limiting	Root	75.91	30082 (−1.9)	21509 (6.1)	13713 (20)
SRG150	Limiting	Root	79.40	29935 (−2.8)	21745 (4.3)	13962 (9)
SRG100	Sufficient	Leaf	70.09	29004	17729	10005
SRG200	Sufficient	Leaf	64.21	28124	16620	8764
SRG150	Sufficient	Leaf	69.75	28915	17220	8984
SRG100	Sufficient	Root	73.33	30381	19679	10777
SRG200	Sufficient	Root	75.52	30661	20269	11475
SRG150	Sufficient	Root	76.08	30801	20840	12758

### Identification of differentially expressed genes in leaves and roots of the three genotypes under limiting N conditions

Pairwise comparisons were made within root or leaf tissue of each genotype between the sufficient and low nitrogen conditions. Differentially expressed (DE) genes were identified if the FPKM for a gene was greater than 1 in at least one of the two conditions being compared and the p-value after adjusting for false discovery was less than 0.05. With these criteria, 688, 322, and 643 genes were significantly differentially expressed in SRG100, SRG200, and SRG150 leaves, with 163, 134, 253 up-regulated and 525, 188, 390 down-regulated under N limitation, respectively (Table 2). In the roots, 675, 585, and 725 genes were significantly differentially expressed in SRG100, SRG200, and SRG150, with 237, 246, and 184 up-regulated and 438, 339, and 541 down-regulated, respectively, under N limiting conditions (Table 2, Additional file 1). As the SRG200 genome has more similarity to the B73 genome, the expression of some SRG200 DE genes (both up-regulated and down-regulated) were selected and tested by qRT-PCR. The results verified what we observed from the RNA_seq data (Additional file 2). Gene Ontology (GO) functional enrichment analysis was performed using Singular Enrichment Analysis (SEA) on AgriGO [25] with the 12 gene lists from each of the six sufficient N to low N pairwise comparisons (Additional file 1 with the up-regulated and down-regulated genes separated as two lists). A cross comparison of SEA (SEACOMPARE) on AgriGO [25] was then performed to compare the GO terms enriched either in leaf or in root for up-regulated or down-regulated genes. In leaf samples, 10 GO terms were enriched in the list of genes up-regulated in response to N limitation for SRG100. Two of these terms were organic acid transport (GO:0015849) and carboxylic acid transport (GO:0046942) (Additional file 3). None were enriched in the up-regulated gene list for SRG200. For SRG150, 31 GO terms were enriched in the up-regulated gene list, including many biological processes important for N metabolism such as the cellular nitrogen compound metabolic process (GO:0034641) and the cellular amino acid metabolic process (GO:0006520) (Additional file 3). 34 GO terms were over-represented in the down-regulated gene list for SRG100, including photosynthesis (GO:0015979); photosynthesis, light harvesting (GO:0009765); photosynthesis, light reaction (GO:0019684); cellular nitrogen compound metabolic process (GO:0034641) and the polysaccharide metabolic process (GO:0005976) (Additional file 3). Four were enriched in the down-regulated gene list for SRG200. 25 GO terms were enriched in the down-regulated gene list for SRG150, including GO terms enriched for SRG100 such as the carbohydrate metabolic process (GO:0005975), and some GO terms were only over-represented in the hybrid, such as the response to stress (GO:0006950) and to abiotic stimulus (GO:0009628) (Additional file 3).

**Table 2:** Significantly differentially expressed (DE) genes identified

**Genotype**	**Tissue**	**Comparison**	**# Differentially expressed genes (**↓ **down-regulated,** ↑ **up-regulated)**
SRG100	Leaf	Sufficient N vs. Limiting N	688 (525 ↓, 163 ↑)
SRG200	Leaf	Sufficient N vs. Limiting N	322 (188 ↓, 134 ↑)
SRG150	Leaf	Sufficient N vs. Limiting N	643 (390 ↓, 253 ↑)
SRG100	Root	Sufficient N vs. Limiting N	675 (438 ↓, 237 ↑)
SRG200	Root	Sufficient N vs. Limiting N	585 (339 ↓, 246 ↑)
SRG150	Root	Sufficient N vs. Limiting N	725 (541 ↓, 184 ↑)

In roots, 18, 26, and 23 GO terms were enriched, respectively, among genes up-regulated in response to N limitation for SRG100, SRG200, and SRG150, with some of these enriched in all three genotypes (Additional file 4). Some GO terms were only enriched in the two parents or in SRG200 and SRG150, and other GO terms were enriched only in SRG150, such as anion transport GO:0006820) and ion transport (GO:0006811) (Additional file 4). 20, 45, and 47 GO terms were enriched respectively in the genes down-regulated in response to N limitation for SRG100, SRG200, and SRG150. The terms photosynthesis (GO:0015979); photosynthesis, light harvesting (GO:0009765); photosynthesis, light reaction (GO:0019684) were down-regulated in all three genotypes, although the number of genes enriched in these groups was different, with the hybrid having the smallest number (Additional file 4). Again, some GO terms were enriched in the two parents such as generation of precursor metabolites and energy (GO:0006091), or in SRG200 and SRG150, such as gene expression (GO:0010467) and cellular macromolecule biosynthetic process (GO:0034645) (Additional file 4). Other GO terms were enriched only in SRG150 such as regulation of gene expression (GO:0010468), regulation of primary metabolic process (GO:0080090), and regulation of nitrogen compound metabolic process (GO:0051171) (Additional file 4).

**Figure 1:**
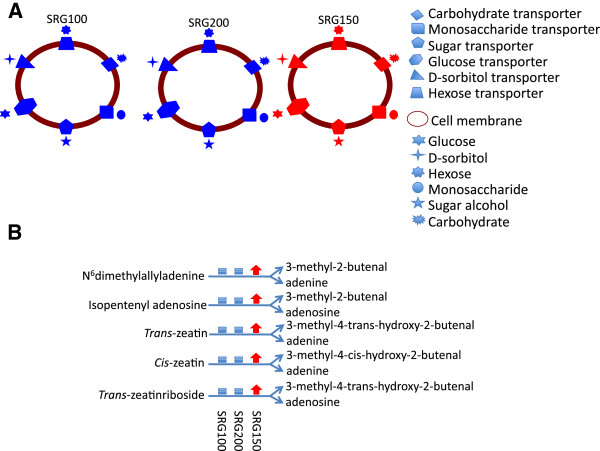
**Highlight of pathways where N-responsive genes in leaves of the three genotypes are different: (A) Sugar transporter pathways; (B) Cytokinin degradation pathways.** In red: up-regulated; in blue: not significantly changed.

To have an overview of the major differences among these differentially expressed genes in the three genotypes, we first took the differentially expressed gene lists in leaves and uploaded these to the Pathway Tools Omics Viewers from the GRAMENE website (http://pathway.gramene.org/expression.html). It is clear that many different pathways were involved even with the limited set of differentially expressed genes (Additional file 5). Some examples include the sugar transporter, carbohydrate transporter, monosaccharide transporter, glucose transporter and sorbitol transporter pathways which were up-regulated in SRG150 under N limitation, but not in SRG100 and SRG200 (Figure 1A). Also of note was the up-regulation of the cytokinin degradation pathway in SRG150, but not in the two parental lines (Figure 1B).

**Figure 2:**
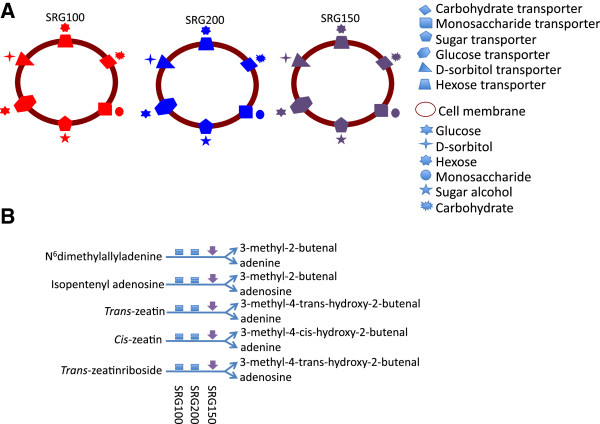
**Highlight of pathways where N-responsive genes in roots of the three genotypes different: (A) Sugar transporter pathways; (B) Cytokinin degradation pathways.** In red: up-regulated; in purple: down-regulated; in blue: not significantly changed.

The differentially expressed gene lists in roots were also uploaded to the Pathway Tools Omics Viewers. The patterns in the roots differed from those in the leaves (Additional file 6). As an example, the sugar transporter, carbohydrate transporter, and monosaccharide transporter pathways were up-regulated in SRG100 under N limitation, not significantly changed in SRG200, but were down-regulated in SRG150 (Figure 2A). Also, the cytokinin degradation pathway was down-regulated in SRG150 roots (Figure 2B).

### Assessment of additive expression in the hybrid

**Figure 3:**
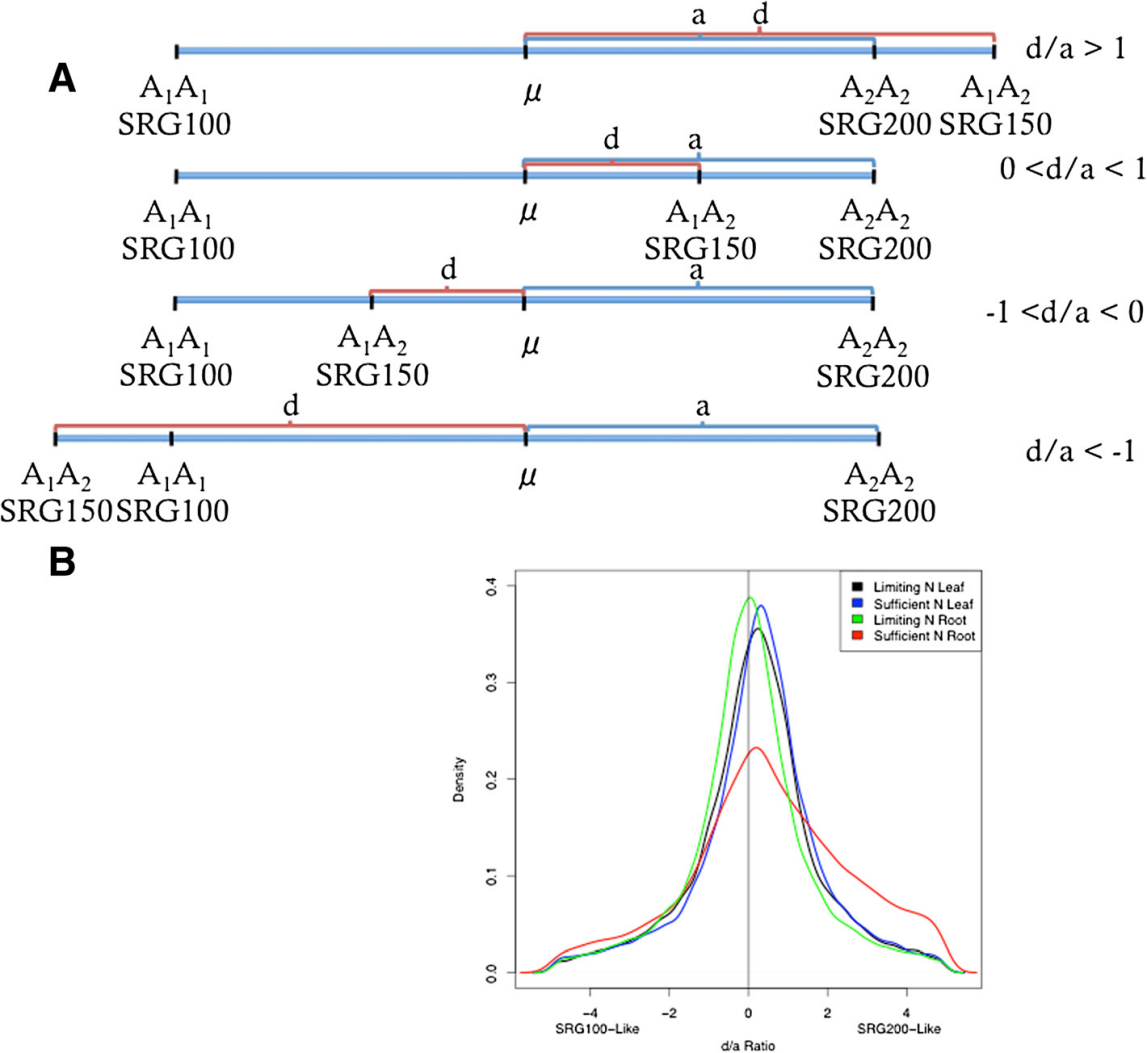
**Assessment of hybrid expression additivity** ***: *** **(A) Schematic diagram of potential patterns of hybrid gene expression; (B) Results of using the d/a ratio to quantify the level of deviation in transcript expression of the hybrid relative to the midparent value of SRG100 and SRG200.**

One of the primary purposes of this study was to determine if transcript levels in the hybrid resembled one inbred more than the other. The dominance-to-additive effects (d/a) ratio is conventionally used to compare trait values between hybrids and inbreds to determine the mode of inheritance. We used a modified version of this ratio as described in Guo et al. [26] to compare hybrid transcript expression levels relative to levels in SRG100 and SRG200 to determine if overall gene expression levels in the hybrid resemble one parent over the other. Details are described in the Methods section. Schematic diagrams of potential patterns of hybrid gene expression are shown in Figure 3A.

The Wilcoxon signed rank test was used to test if the mean of genes’ d/a ratios significantly deviated from 0. The results showed that transcript expression levels in the hybrid were significantly skewed towards SRG200 (Table 3, Figure 3B). Genes were divided into 16 lists: genes with d/a ratios below −1, genes with d/a ratios between −1 and 0, genes with d/a ratios between 0 and 1, and genes with d/a ratios above 1, respectively, for low N in leaves, low N in roots, sufficient N in leaves, and sufficient N in roots. Gene Ontology functional enrichment analysis was performed using AgriGO [25] on the 16 gene lists individually (Additional file 7). SEAcompare by AgriGO was performed to contrast the GO terms enriched under different N conditions either in leaf or in root. It is clear from the analysis that leaf and root tissue responded differently to N limitation. Many more GO terms were enriched in the group of genes with expression levels skewed towards SRG200 under N limitation in leaf while more GO terms were enriched in roots under sufficient N conditions (Table 3). It is also clear that there are different expression patterns for certain groups of genes under the two N conditions (Additional files 8 and 9). Expression patterns in the hybrid can resemble one parent in one N treatment and the other parent in another N treatment. The examples for these include GO:0009628 (response to abiotic stimulus), GO:0016051 (carbohydrate biosynthetic process), and GO:0000271 (polysaccharide biosynthetic process), which is clearly seen in leaf (Additional file 8), but not in root (Additional file 9). Some GO terms were enriched under sufficient N in the group of genes with hybrid expression levels similar to either SRG100 and SRG200 but were only enriched in the group of genes with hybrid expression levels skewed towards SRG200 under low N conditions, e.g. photosynthesis (GO:0015979), photosynthesis light harvesting (GO:0009765) in leaf (Additional file 8), and cellular carbohydrate metabolic process (GO:0044262) in root (Additional file 9). Some groups of genes in the hybrid were expressed at levels between midparent and SRG200 (0 < d/a < 1) under sufficient N condition, but had more genes expressed at levels outside the SRG200 range under low N condition (d/a >1), e.g. gene expression (GO:0010467) in leaf (Additional file 8), and carbohydrate metabolic process (GO:0005975) in root (Additional file 9). Some GO terms were only enriched under low N conditions with expression skewed towards SRG200 such as phosphate metabolic process (GO:0006796), cellular amino acid metabolic process (GO:0006520), photosynthesis, light reaction (GO:0019684) in leaf (Additional file 8), while some GO terms were only enriched under sufficient N conditions with expression skewed towards SRG200 such as cellular amino acid metabolic process (GO:0006520), glutamine family amino acid metabolic process (GO:0009064) in root (Additional file 9). As we know from our previous results under N limitation SRG150 responded more similarly to SRG200 rather than SRG100 and glutamine metabolic process always plays an important role in NUE; these results support the notion that the differences in expression correlate well with the differences in the NUE traits in these lines.

**Table 3:** Expression in the hybrid under different N conditions and GO terms enriched

**N condition**	**Tissue**	**d/a < −1**	**0 > d/a > −1**	**1 > d/a > 0**	**d/a > 1**
Number of genes					
Sufficient	Leaf	3990	3595	5773	5818
Limiting	Leaf	4489	4281	5784	5919
Sufficient	Root	5765	2897	3454	9979
Limiting	Root	5372	6093	6182	5672
GO terms enriched (with BP, MF, CC)					
Sufficient	Leaf	56	28	62	63
Limiting	Leaf	15	26	67	86
Sufficient	Root	42	10	52	147
Limiting	Root	24	51	38	30
d/a < −1		expression level outside range of SRG100			
0 > d/a > −1		expression level skewed towards SRG100			
1 > d/a > 0		expression level skewed towards SRG200			
d/a > 1		expression level outside range of SRG200			

BP: biological process.

MF: molecular function.

CC: cellular component.

### Identification of allelic expression in the hybrid

**Figure 4:**
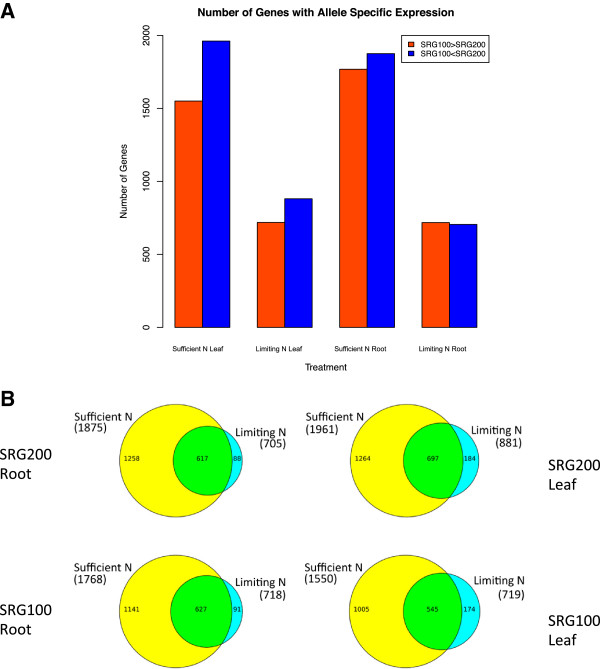
**Allelic expression in the hybrid: (A) Number of genes expressing different parental alleles under sufficient or limiting N conditions in leaves or roots; (B) Venn diagrams of genes expressing different parental alleles under sufficient or limiting N conditions in leaves or roots using Findpeaks 3.1 **[27]**.**

As parental genetic diversity serves as the basis of heterosis, we investigated genes for which the level of expression from each parental allele differed in the hybrid. To do this, we assembled *de novo* transcriptomes for SRG100 and SRG200 and called SNPs between the transcriptomes. 67,760 SNPs were found and used to determine differential allele expression in the hybrid at FGS genes with mean read depths greater than 10 reads per SNP in the hybrid sample. The number of genes with differential allele expression varied depending on tissue and nitrogen status (Figure 4A). In general, a higher percentage of genes exhibited differential allele expression under the sufficient nitrogen condition in both leaves and roots. SRG200 alleles tended to be consistently more highly expressed than SRG100 alleles in leaves but not in roots. Finally, a large proportion of genes showing differential allele expression in the low nitrogen sample also showed this in the sufficient nitrogen sample (Figure 4B).

In root samples both parental alleles had similar probabilities of exhibiting the more highly expressed allele for genes with differential allele expression. However, the GO terms represented from the sets of parental alleles differ significantly (Additional file 10). The details of these genes are listed in Additional file 11. In leaves, the cellular N compound metabolic process (GO:0034641) was enriched for the hybrid regardless of which parental allele was more highly expressed under sufficient N condition, but was only enriched in the hybrid when the SRG200 allele was more highly expressed under the low N condition (Additional file 10). The hexose metabolic process (GO:0019318) and glucose metabolic process (GO:0006006) were enriched in the hybrid when expressing SRG200 alleles more highly than SRG100 alleles under sufficient N condition (Additional file 10). In the roots, the enriched GO terms were quite different from the ones in the shoots although the cellular N compound metabolic process (GO:0034641) was enriched in the hybrid when the allele from either SRG100 or SRG200 was more highly expressed under sufficient N condition, but only enriched in the hybrid when SRG200 alleles were more highly expressed than SRG100 alleles under low N condition, which was similar in the shoots (Additional file 10).

## Discussion

### Transcriptome changes under N limitation for the three genotypes shows different mechanisms to deal with N limitation

Efforts have been directed to understand the mechanisms of how plants respond to N limitation. Many approaches have been used, and one of these is transcriptome profiling [6]. Microarray technology has been used in the past for analyzing genome-scale gene expression [28]. Extensive studies have been performed for *Arabidopsis thaliana*[29-39]. There have also been studies for various crops such as rice seedlings at an early stage of low N stress [40] and the model legume *Medicago truncatula*[41]. Recently, Yang et al. [42] utilized multiple whole-genome microarray experiments to identify gene expression biomarkers in maize, which can be used to monitor nitrogen status. The microarray technology, however, has a few intrinsic limitations. The dynamic range of microarrays is restricted by factors such as the probe density/availability and the intensities of fluorescent dyes, as well as reduced sensitivity by non-specific cross-hybridization which can mask isoform expression and inflate the expression of rare transcripts [43]. One significant advantage of sequence-based transcriptomics is the potential to precisely quantify the abundance of any transcript, drastically increasing the dynamic range of the experiment [44]. Considering the advantages, we did a survey of the maize transcriptome using the mRNA**-**Seq technique for two parental inbred lines and the corresponding hybrid line, for which a number of phenotypic, molecular, and metabolic factors were previously studied under sufficient and limiting N conditions [22].

From our results, it is evident that the dynamic changes in the transcriptome for the three genotypes reflect the differences in their response to growth under limiting N. Between the two parents, SRG200 demonstrated a better strategy to deal with N limitation, and the hybrid was superior to the parents [22]. From the DE genes identified and the GO terms enriched, the different responses are noticeable among the three genotypes (Additional files 3 and 4; Figures 1 and 2). In leaf tissues, the hybrid shows an enhancement in the cellular nitrogen compound metabolic process, the cellular amino acid metabolic process, and transport when the plants were under N stress, and these changes were not seen in the parental lines. Although the hybrid showed a reduction in the cellular carbohydrate metabolic process under N limitation, the genes involved in photosynthesis were not over-represented in the down-regulated gene list, which was different from the SRG100 parental line, suggesting that the photosynthesis rate was not down-regulated as much in the hybrid as in the SRG100 parental line (Additional file 3). As C and N metabolism are closely linked and tightly regulated [45,46], maintaining an adequate photosynthetic rate would certainly favor an efficient production of reduced C and the subsequent efficient use of N. This result correlates well with our physiological tests in a previous study where SRG200 and SRG150 maintained higher sugar content in leaves than SRG100 [22]. In root tissues, both the hybrid and the SRG200 parent significantly increased transport activity, which was not seen in the SRG100 parental line, and the down-regulation of gene expression associated with primary metabolism was very significant in the hybrid (Additional file 4). It has been well documented that root/shoot ratios would increase when plants are grown under N-limiting conditions [47] and that there is an interaction between nitrogen and cytokinin [48]. Interestingly, the cytokinin degradation pathway was up-regulated in the shoots and down-regulated in the roots under N limitation only in the hybrid (Figures 1 and 2). Less reduction of root biomass in the hybrid under N limitation was observed from our previous study [22], and the down-regulation of the cytokinin degradation in the hybrid roots under N limitation might be one of the mechanisms for the hybrid to adapt to N limitation. The limited expression data from our previous study suggested that the three genotypes had different mechanism to cope with N stress [22]. The present transcriptome data supports that former observation as the three genotypes presented a different enriched gene set when they had to deal with N stress.

### The change in gene expression in the hybrid resembles one parent with a similar NUE trait under N limitation

From our previous study, we learned that the parental line SRG200 had higher NUE than SRG100 and that heterosis was observed in the hybrid SRG150 [22]. In this study, we found that there was a dynamic reprogramming of the transcriptome and the hybrid gene expression levels were significantly more similar to SRG200 levels than SRG100 levels when the hybrid plants were experiencing N limitation (Table 3, Additional files 8 and 9, Figure 3B). This result demonstrated that the transcriptomic similarity mimicked phenotypic similarity.

One possible explanation for the similarity in changes between gene expression levels of one of the parents, SRG200, and the hybrid would be that some alleles derived from that parent control expression of other genes in the hybrid, particularly under the N limiting condition. Across all genes SRG200 alleles were slightly more likely to be up-regulated than SRG100 alleles but the difference was not statistically significant. Expression level is determined by a combination of *cis*-acting and *trans*-acting regulatory sequences. Changes in the expression of the latter, which might be allele specific, would lead to changes in expression of a variety of the regulated genes in a non-allele specific manner. Further investigation is needed to understand how plants sense N limitation and change the inventories of the expression of allele-specific genes and how this correlates with the NUE trait in different genotypes.

## Conclusions

Gene expression under N limitation in two parental inbreds and the corresponding hybrid line that responded differently to N limitation was surveyed using the mRNA-Seq technology. The data showed that the three genotypes have different mechanisms to deal with N-limiting conditions. Gene expression levels are correlated with the ability of a particular line to respond to growth under limiting N. There was allele-specific expression in the hybrid with a slight bias to the parent that grew better under limiting N. This study enhances our current understanding of the response to growth under N limitation, and the results of this type of study can be used to develop plants with improved NUE.

## Methods

### Plant materials and growth condition

The plant materials and growth conditions were identical to our previous study [22]. Briefly, seeds of the two elite maize inbred lines, SRG100 and SRG200, and the hybrid line, SRG150, created by crossing the two inbred lines, (Syngenta Biotechnology Inc. NC, USA), were germinated in turface for 2 days, and then transplanted to the hydroponic system in nutrient solution containing 4 mM MgSO_4_, 5 mM KCl, 5 mM CaCl_2_, 1 mM KH_2_PO_4_, 0.1 mM Fe-EDTA, 0.5 mM MES (pH 6.0), 9 μM MnSO_4_, 0.7 μM ZnSO_4_, 0.3 μM CuSO_4_, 46 μM NaB_4_O_7_ and 0.2 μM (NH_4_)6Mo_7_O_2_. Seedlings were transferred to a 35 L container containing 25 L of the nutrient solution; the volume and the pH were adjusted weekly by adding fresh nutrient solution and using phosphoric acid to adjust the pH to 5.5. Two different nitrate (KNO_3_) concentrations were used; one as sufficient N condition (3 mM) and one as limiting N condition (1 mM) [22]. Plants were grown in a growth cabinet (Conviron, Manitoba, Canada) under long day conditions of 16 hr light (~500 μmol m^-2^ s^-1^) at 28°C and 8 hr dark at 23°C. Plants were harvested four weeks later. Leaves (3rd to 5th) and the whole roots were collected separately. Plant harvest was carried out at noon for each sample which was pooled from 2–3 plants. The materials were submerged in RNAlater (Ambion Inc., TX, USA) and stored at −80°C until further analysis.

### RNA extraction, quality control, normalization, mRNA Seq library construction and Illumina SBS

mRNA was extracted using mirVana™ miRNA isolation kit (Ambion Inc., TX, USA). Using a Bio-Rad Experion system (Hercules, CA, USA), total RNA integrity was measured. An RNA Quality Index (RQI) value greater than 8 was selected as the cut-off value for the total RNA quality control. The RNA samples that passed the QC process were used in the mRNA-Seq library construction. Following the Illumina manual of Preparing Samples for Sequencing of mRNA (Illumina, San Diego, CA, USA), 5 ug of total RNA for each sample were used in the mRNA-Seq library construction. Sera-mag Magnetic Oligo(dT) beads were used to purify the poly-A containing mRNA molecules. Subsequently the purified mRNA was fragmented into small pieces using divalent cations under elevated temperature, and reverse transcribed into cDNA using SuperScript II (Invitrogen, Carlsbad, CA, USA). The cDNA went through an end repair process, the addition of a single ‘A’ base to the 3′ ends, and ligation of the Illumina paired-end sequencing adapters. The ligation products were fragmented on a 2% agarose TAE gel, and the gel slices containing material in the 200 bp (±15 bp) range were excised. cDNA was purified from the gel slices using QIAquick Gel Extraction Kit (QIAGEN, Valencia, CA). Finally, the size-selected cDNA libraries ligated to the Illumina sequencing adaptors were selectively enriched using 15 cycles of PCR, and validated using a Bio-Rad Experion system. Each final cDNA library was then applied on one lane of the Illumina paired-end flow cell for the cluster generation process and subsequently sequenced using the Illumina next-generation sequencing platform GA II as 2 × 36 or 2 × 40 bp paired-end reads.

### Sequenced read processing and alignment

Reads were aligned to the B73 reference genome version 2 (maizesequence.org) using Tophat v1.4.1 [49] and Bowtie v0.12.7 [50]. Before alignment, Bowtie quality control removed 0.1-0.2% of the total reads. A minimum intron length of 5 and a maximum intron length of 5000 were used for alignment. Segment lengths were set to half the read lengths and segment mismatches were set to 1. All other parameters were set to default.

### Identification of expressed genes

A reference annotation from Ensembl Genomes (zea_mays.AGPv2.62.gtf) was used to guide transcript assembly by Cufflinks v1.3.0 [51] to obtain fragments per kilobase of exon per million fragments mapped (FPKM) for all genes within the WGS**.** Fragment bias correction [52], which corrects for sequence-specific bias, and multi-hit read correction, which divides the value of a multi-mapped read between each map location based on a probabilistic model, were used with Cufflinks. Cuffmerge was used to create a single unified assembly from each of the 12 individual Cufflinks assemblies. Cuffmerge maximizes assembly quality by removing transcripts that are artifacts and merging novel isoforms with known isoforms across all Cufflinks assemblies. A transcript was considered to be expressed if its FPKM value was greater than one and if it was part of the maize Filtered Gene Set (FGS) version 5b.60 (maizesequence.org). The FGS is a list of maize genes in which pseudogenes, transposable element encoding genes, and low-confidence hypothetical models have been removed.

### Identification of significantly differentially expressed (DE) genes

Cufflinks [51] was used to perform pairwise comparisons between samples to find differentially expressed transcripts. Fragment bias correction [52], multi-hit read correction, and upper-quartile normalization [53], which causes Cufflinks to divide the number of reads mapped to each gene by 75th quartile of the counts instead of dividing by the total number of mapped reads for normalization, were used with Cufflinks. An FGS transcript was differentially expressed between samples if the FPKM in one sample was greater than one and if p-value after correcting for multiple testing with the Benjamini-Hochberg correction was less than 0.05.

### d/a analysis

The d/a ratio (Eq. 1) was used to quantify the level of deviation in transcript expression of SRG150 relative to the midparent value of SRG100 and SRG200 [26] for any genes with FPKM > 1 in SRG100, SRG200, or SRG150.

$$\frac{d}{a}=\frac{F_{1}-\mu }{P_{1}-\mu }$$

In the equation, F_1_ is the transcript expression level in SRG150, μ is the average gene expression level in the two inbred parents, P_1_ is the gene expression level in SRG200. If F_1_ = P_1_, then d/a = 1 and the gene shows dominant gene action from the SRG200 allele. Genes with d/a values between −1 < d/a < 0 exhibit hybrid expression levels skewed towards SRG100 levels, and genes with d/a values between 0 < d/a < 1 exhibit hybrid expression levels skewed towards SRG200. Genes with d/a values greater than 1.0 or less than −1.0 have hybrid expression levels outside of the parental range. Genes with d/a values of 0 have expression levels in the hybrid that are additive and favor neither parent. The one sample Wilcoxon test [54] was used on the d/a estimates to determine if hybrid transcript expression across all genes deviated significantly from expected additive parental levels and to determine the overall direction of bias.

### Identification of SNPs

Trinity [55] was used to create *de novo* transcriptomes for SRG100 and SRG200. The contigs from the *de novo* transcriptomes were aligned to the B73 reference genome to find common contigs between the two transcriptomes and to call SNPs between the two transcriptomes. The hybrid mRNA-Seq reads were aligned separately to both transcriptomes and read depths were determined using SamTools [56] at 67,760 SNPs. SRG100 allele depths were estimated from hybrid reads aligned to the SRG100 transcriptome, and SRG200 allele depths were estimated from hybrid reads aligned to the SRG200 transcriptome. For a read to count towards the allele depth of a given parent, it needed to match the base at the SNP position for the given parent. FGS genes with mean SNP read depths greater than 10 reads per SNP in the gene were used for allelic imbalance analysis. The binomial exact test with an alpha value of 0.05 was used to determine if a gene had preferential expression for the allele of one parent over that of the other parent.

### Availability of supporting data

The datasets supporting the results of this article are available in the Sequence Read Archive (SRA). The accession ID is SRP033653, with the following link: http://www.ncbi.nlm.nih.gov/sra/?term=SRP033653.

## Competing interests

The authors declare that they have no competing interests.

## Authors’ contributions

YMB conceived the project, analyzed the data and wrote the manuscript. AM analyzed the RNA_seq data and helped write manuscript on the analysis of the data. GSD helped analyzing the RNA_seq data and editing the manuscript. XJS did the RNA sequencing and initial data analysis. AE developed conditions for plant growth and prepared samples. LL supervised data analysis and manuscript writing. SJR supervised the project, data analysis and contributed to manuscript writing. All authors read and approved the final manuscript.

## Supplementary Material

Additional file 1 Significantly differentially expressed genes in leaves and roots of the three genotypes. Click here for file

Additional file 2 Expression of selected DE genes verified by qRT-PCR. Click here for file

Additional file 3 Selected significantly enriched biological processes in the leaves of the three genotypes under N limitation. Click here for file

Additional file 4 Selected significantly enriched biological processes in the roots of the three genotypes under N limitation. Click here for file

Additional file 5 Overview of pathways where N-responsive genes in leaves of the three genotypes involved. Click here for file

Additional file 6 Overview of pathways where N-responsive genes in roots of the three genotypes involved. Click here for file

Additional file 7 Different groups of genes with expression levels in leaves and roots skewingtowards one parent or another under sufficient or limiting N conditions. Click here for file

Additional file 8 Selected significantly enriched GO terms in leaves under sufficient or limiting N conditions. Click here for file

Additional file 9 Selected significantly enriched GO terms in roots under sufficient or limiting N conditions. Click here for file

Additional file 10 Selected enriched GO terms in the hybrid expressing SRG100 or SRG200 alleles under different N conditions. Click here for file

Additional file 11 Allelic expression in the leaves or roots of the hybrid under sufficient or limiting N conditions. Click here for file
