# Supplementary material for: High throughput RNA sequencing of a hybrid maize and its parents shows different mechanisms responsive to nitrogen limitation
Source: BMC Genomics. 2014 Jan 28;15:77. doi: 10.1186/1471-2164-15-77 (PMC3912931; doi:10.1186/1471-2164-15-77)
Supplement: Additional file 3 — Selected significantly enriched biological processes in the leaves of the three genotypes under N limitation. [file 1471-2164-15-77-S3.doc]

| **Additional file 3. Selected significantly enriched biological processes in the leaves of the three genotypes under N limitation** | | | | | | | | | | | |
| --- | --- | --- | --- | --- | --- | --- | --- | --- | --- | --- | --- |
|  |  |  |  |  |  |  |  |  |  |  |  |
|  | | |  | | | **SRG100** | | **SRG200** | | **SRG150** | |
| **GO Term** | **Onto** | **Description** | **SRG100** | **SRG200** | **SRG150** | **FDR** | **Num** | **FDR** | **Num** | **FDR** | **Num** |
|  |  |  |  |  |  |  |  |  |  |  |  |
| **Up-regulation** |  |  |  |  |  |  |  |  |  |  |  |
| GO:0015849 | P | organic acid transport |  |  |  | 0.0014 | [6](http://bioinfo.cau.edu.cn/agriGO/termDetail.php?session=822817074&GO=GO:0015849) | --- | --- | --- | --- |
| GO:0046942 | P | carboxylic acid transport |  |  |  | 0.0014 | [6](http://bioinfo.cau.edu.cn/agriGO/termDetail.php?session=822817074&GO=GO:0046942) | --- | --- | --- | --- |
| GO:0009072 | P | aromatic amino acid family metabolic process |  |  |  | --- | --- | --- | --- | 0.0025 | [6](http://bioinfo.cau.edu.cn/agriGO/termDetail.php?session=246894673&GO=GO:0009072) |
| GO:0034641 | P | cellular nitrogen compound metabolic process |  |  |  | --- | --- | --- | --- | 0.0036 | [11](http://bioinfo.cau.edu.cn/agriGO/termDetail.php?session=246894673&GO=GO:0034641) |
| GO:0006520 | P | cellular amino acid metabolic process |  |  |  | --- | --- | --- | --- | 0.043 | [10](http://bioinfo.cau.edu.cn/agriGO/termDetail.php?session=246894673&GO=GO:0006520) |
| GO:0044106 | P | cellular amine metabolic process |  |  |  | --- | --- | --- | --- | 0.043 | [10](http://bioinfo.cau.edu.cn/agriGO/termDetail.php?session=246894673&GO=GO:0044106) |
| GO:0030001 | P | metal ion transport |  |  |  | --- | --- | --- | --- | 0.043 | [9](http://bioinfo.cau.edu.cn/agriGO/termDetail.php?session=246894673&GO=GO:0030001) |
|  |  |  |  |  |  |  |  |  |  |  |  |
|  |  |  |  |  |  |  |  |  |  |  |  |
| **Down-regulation** | |  |  |  |  |  |  |  |  |  |  |
| GO:0015979 | P | Photosynthesis |  |  |  | 1.20E-08 | [16](http://bioinfo.cau.edu.cn/agriGO/termDetail.php?session=393656829&GO=GO:0015979) | --- | --- | --- | --- |
| GO:0009765 | P | photosynthesis, light harvesting |  |  |  | 2.30E-03 | [5](http://bioinfo.cau.edu.cn/agriGO/termDetail.php?session=393656829&GO=GO:0009765) | --- | --- | --- | --- |
| GO:0019684 | P | photosynthesis, light reaction |  |  |  | 0.0061 | [6](http://bioinfo.cau.edu.cn/agriGO/termDetail.php?session=393656829&GO=GO:0019684) | --- | --- | --- | --- |
| GO:0005976 | P | polysaccharide metabolic process |  |  |  | 0.00094 | [13](http://bioinfo.cau.edu.cn/agriGO/termDetail.php?session=393656829&GO=GO:0005976) | --- | --- | --- | --- |
| GO:0034641 | P | cellular nitrogen compound metabolic process |  |  |  | 0.045 | [13](http://bioinfo.cau.edu.cn/agriGO/termDetail.php?session=393656829&GO=GO:0034641) | --- | --- | --- | --- |
| GO:0005975 | P | carbohydrate metabolic process |  |  |  | 0.000022 | [38](http://bioinfo.cau.edu.cn/agriGO/termDetail.php?session=393656829&GO=GO:0005975) | --- | --- | 0.0000027 | [33](http://bioinfo.cau.edu.cn/agriGO/termDetail.php?session=424714513&GO=GO:0005975) |
| GO:0006950 | P | response to stress |  |  |  | --- | --- | --- | --- | 0.0039 | [37](http://bioinfo.cau.edu.cn/agriGO/termDetail.php?session=424714513&GO=GO:0006950) |
| GO:0042592 | P | homeostatic process |  |  |  | --- | --- | --- | --- | 0.021 | [28](http://bioinfo.cau.edu.cn/agriGO/termDetail.php?session=424714513&GO=GO:0042592) |
| GO:0009628 | P | response to abiotic stimulus |  |  |  | --- | --- | --- | --- | 0.036 | [24](http://bioinfo.cau.edu.cn/agriGO/termDetail.php?session=424714513&GO=GO:0009628) |
|  |  |  |  |  |  |  |  |  |  |  |  |
|  |  |  |  |  |  |  |  |  |  |  |  |
| The analysis was performed using the Singular Enrichment Analysis (SEAcompare) on the AgriGO website (Du et al., 2010, http://bioinfo.cau.edu.cn/agriGO/). | | | | | | | | | | | |
| This tool allowed the identification of GO terms that were significantly enriched in the lists of entities differentially regulated by N limitation. | | | | | | | | | | | |
| For each genotype, the false discovery rate (FDR) and the number of entities (Num) are shown where the GO term enrichment was significant. | | | | | | | | | | | |
| In those cases, the cells in the table are filled with increasing shades of red as the FDR decreases. | | | | | | | | | | | |
| Only some of the GO terms involved in the biological process (P) are presented here. | | | | | | | | | | | |
