# Supplementary material for: High throughput RNA sequencing of a hybrid maize and its parents shows different mechanisms responsive to nitrogen limitation
Source: BMC Genomics. 2014 Jan 28;15:77. doi: 10.1186/1471-2164-15-77 (PMC3912931; doi:10.1186/1471-2164-15-77)
Supplement: Additional file 4 — Selected significantly enriched biological processes in the roots of the three genotypes under N limitation. [file 1471-2164-15-77-S4.doc]

| **Additional file 4. Selected significantly enriched biological processes in the roots of the three genotypes under N limitation** | | | | | | | | | | | | |
| --- | --- | --- | --- | --- | --- | --- | --- | --- | --- | --- | --- | --- |
|  |  |  |  |  |  |  |  |  |  |  |  | |
|  | | |  | | | **SRG100** | | **SRG200** | | **SRG150** | | |
| **GO Term** | **Onto** | **Description** | **SRG100** | **SRG200** | **SRG150** | **FDR** | **Num** | **FDR** | **Num** | **FDR** | **Num** | |
|  |  |  |  |  |  |  |  |  |  |  |  | |
| **Up-regulation** |  |  |  |  |  |  |  |  |  |  |  | |
| GO:0006979 | P | response to oxidative stress |  |  |  | 1.80E-09 | [16](http://bioinfo.cau.edu.cn/agriGO/termDetail.php?session=294409622&GO=GO:0006979) | 2.00E-07 | [14](http://bioinfo.cau.edu.cn/agriGO/termDetail.php?session=602970528&GO=GO:0006979) | 2.40E-06 | [12](http://bioinfo.cau.edu.cn/agriGO/termDetail.php?session=623170456&GO=GO:0006979) |  |
| GO:0042221 | P | response to chemical stimulus |  |  |  | 0.0000016 | [17](http://bioinfo.cau.edu.cn/agriGO/termDetail.php?session=294409622&GO=GO:0042221) | 5.10E-05 | [15](http://bioinfo.cau.edu.cn/agriGO/termDetail.php?session=602970528&GO=GO:0042221) | 0.000033 | [14](http://bioinfo.cau.edu.cn/agriGO/termDetail.php?session=623170456&GO=GO:0042221) |  |
| GO:0006820 | P | anion transport |  |  |  | --- | --- | --- | --- | 2.30E-02 | [5](http://bioinfo.cau.edu.cn/agriGO/termDetail.php?session=623170456&GO=GO:0006820) |  |
| GO:0006811 | P | ion transport |  |  |  | --- | --- | --- | --- | 5.00E-02 | [13](http://bioinfo.cau.edu.cn/agriGO/termDetail.php?session=623170456&GO=GO:0006811) |  |
|  |  |  |  |  |  |  |  |  |  |  |  |  |
|  |  |  |  |  |  |  |  |  |  |  |  |  |
| **Down-regulation** | |  |  |  |  |  |  |  |  |  |  |  |
| GO:0015979 | P | Photosynthesis |  |  |  | 6.10E-33 | [33](http://bioinfo.cau.edu.cn/agriGO/termDetail.php?session=861288332&GO=GO:0015979) | 2.50E-28 | [28](http://bioinfo.cau.edu.cn/agriGO/termDetail.php?session=608794091&GO=GO:0015979) | 4.00E-06 | [12](http://bioinfo.cau.edu.cn/agriGO/termDetail.php?session=822535423&GO=GO:0015979) |  |
| GO:0009765 | P | photosynthesis, light harvesting |  |  |  | 1.2E-14 | [12](http://bioinfo.cau.edu.cn/agriGO/termDetail.php?session=861288332&GO=GO:0009765) | 5.00E-20 | [14](http://bioinfo.cau.edu.cn/agriGO/termDetail.php?session=608794091&GO=GO:0009765) | 0.0000028 | [7](http://bioinfo.cau.edu.cn/agriGO/termDetail.php?session=822535423&GO=GO:0009765) |  |
| GO:0019684 | P | photosynthesis, light reaction |  |  |  | 9.5E-14 | [14](http://bioinfo.cau.edu.cn/agriGO/termDetail.php?session=861288332&GO=GO:0019684) | 2.8E-15 | [14](http://bioinfo.cau.edu.cn/agriGO/termDetail.php?session=608794091&GO=GO:0019684) | 2.20E-04 | [7](http://bioinfo.cau.edu.cn/agriGO/termDetail.php?session=822535423&GO=GO:0019684) |  |
| GO:0006829 | P | zinc ion transport |  |  |  | 0.0000011 | [5](http://bioinfo.cau.edu.cn/agriGO/termDetail.php?session=861288332&GO=GO:0006829) | 0.00000022 | [5](http://bioinfo.cau.edu.cn/agriGO/termDetail.php?session=608794091&GO=GO:0006829) | --- | --- |  |
| GO:0000041 | P | transition metal ion transport |  |  |  | 0.0017 | [6](http://bioinfo.cau.edu.cn/agriGO/termDetail.php?session=861288332&GO=GO:0000041) | 0.00018 | [6](http://bioinfo.cau.edu.cn/agriGO/termDetail.php?session=608794091&GO=GO:0000041) | --- | --- |  |
| GO:0006091 | P | generation of precursor metabolites and energy |  |  |  | 0.0028 | [17](http://bioinfo.cau.edu.cn/agriGO/termDetail.php?session=861288332&GO=GO:0006091) | 2.70E-04 | [16](http://bioinfo.cau.edu.cn/agriGO/termDetail.php?session=608794091&GO=GO:0006091) | --- | --- |  |
| GO:0010467 | P | gene expression |  |  |  | --- | --- | 0.000001 | [66](http://bioinfo.cau.edu.cn/agriGO/termDetail.php?session=608794091&GO=GO:0010467) | 1.20E-02 | [70](http://bioinfo.cau.edu.cn/agriGO/termDetail.php?session=822535423&GO=GO:0010467) |  |
| GO:0034645 | P | cellular macromolecule biosynthetic process |  |  |  | --- | --- | 0.000003 | [65](http://bioinfo.cau.edu.cn/agriGO/termDetail.php?session=608794091&GO=GO:0034645) | 1.20E-02 | [71](http://bioinfo.cau.edu.cn/agriGO/termDetail.php?session=822535423&GO=GO:0034645) |  |
| GO:0010468 | P | regulation of gene expression |  |  |  | --- | --- | --- | --- | 1.00E-06 | [63](http://bioinfo.cau.edu.cn/agriGO/termDetail.php?session=822535423&GO=GO:0010468) |  |
| GO:0080090 | P | regulation of primary metabolic process |  |  |  | --- | --- | --- | --- | 2.80E-06 | [63](http://bioinfo.cau.edu.cn/agriGO/termDetail.php?session=822535423&GO=GO:0080090) |  |
| GO:0060255 | P | regulation of macromolecule metabolic process |  |  |  | --- | --- | --- | --- | 0.0000028 | [63](http://bioinfo.cau.edu.cn/agriGO/termDetail.php?session=822535423&GO=GO:0060255) |  |
| GO:0051171 | P | regulation of nitrogen compound metabolic process |  |  |  | --- | --- | --- | --- | 3.20E-06 | [60](http://bioinfo.cau.edu.cn/agriGO/termDetail.php?session=822535423&GO=GO:0051171) |  |
| GO:0034641 | P | cellular nitrogen compound metabolic process |  |  |  | --- | --- | --- | --- | 5.90E-03 | [14](http://bioinfo.cau.edu.cn/agriGO/termDetail.php?session=822535423&GO=GO:0034641) |  |
| GO:0050896 | P | response to stimulus |  |  |  | --- | --- | --- | --- | 0.025 | [43](http://bioinfo.cau.edu.cn/agriGO/termDetail.php?session=822535423&GO=GO:0050896) |  |
| The analysis was performed using the Singular Enrichment Analysis (SEAcompare) on the AgriGO website (Du et al., 2010, http://bioinfo.cau.edu.cn/agriGO/). | | | | | | | | | | | | |
| This tool allowed the identification of GO terms that were significantly enriched in the lists of entities differentially regulated by N limitation. | | | | | | | | | | | | |
| For each genotype, the false discovery rate (FDR) and the number of entities (Num) are shown where the GO term enrichment was significant. | | | | | | | | | | | | |
| In those cases, the cells in the table are filled with increasing shades of red as the FDR decreases. | | | | | | | | | | | | |
| Only some of the GO terms involved in the biological process (P) are presented here. | | | | | | | | | | | | |
