# Supplementary material for: High throughput RNA sequencing of a hybrid maize and its parents shows different mechanisms responsive to nitrogen limitation
Source: BMC Genomics. 2014 Jan 28;15:77. doi: 10.1186/1471-2164-15-77 (PMC3912931; doi:10.1186/1471-2164-15-77)
Supplement: Additional file 5 — Overview of pathways where N-responsive genes in leaves of the three genotypes involved. [file 1471-2164-15-77-S5.pptx]

## Slide 1
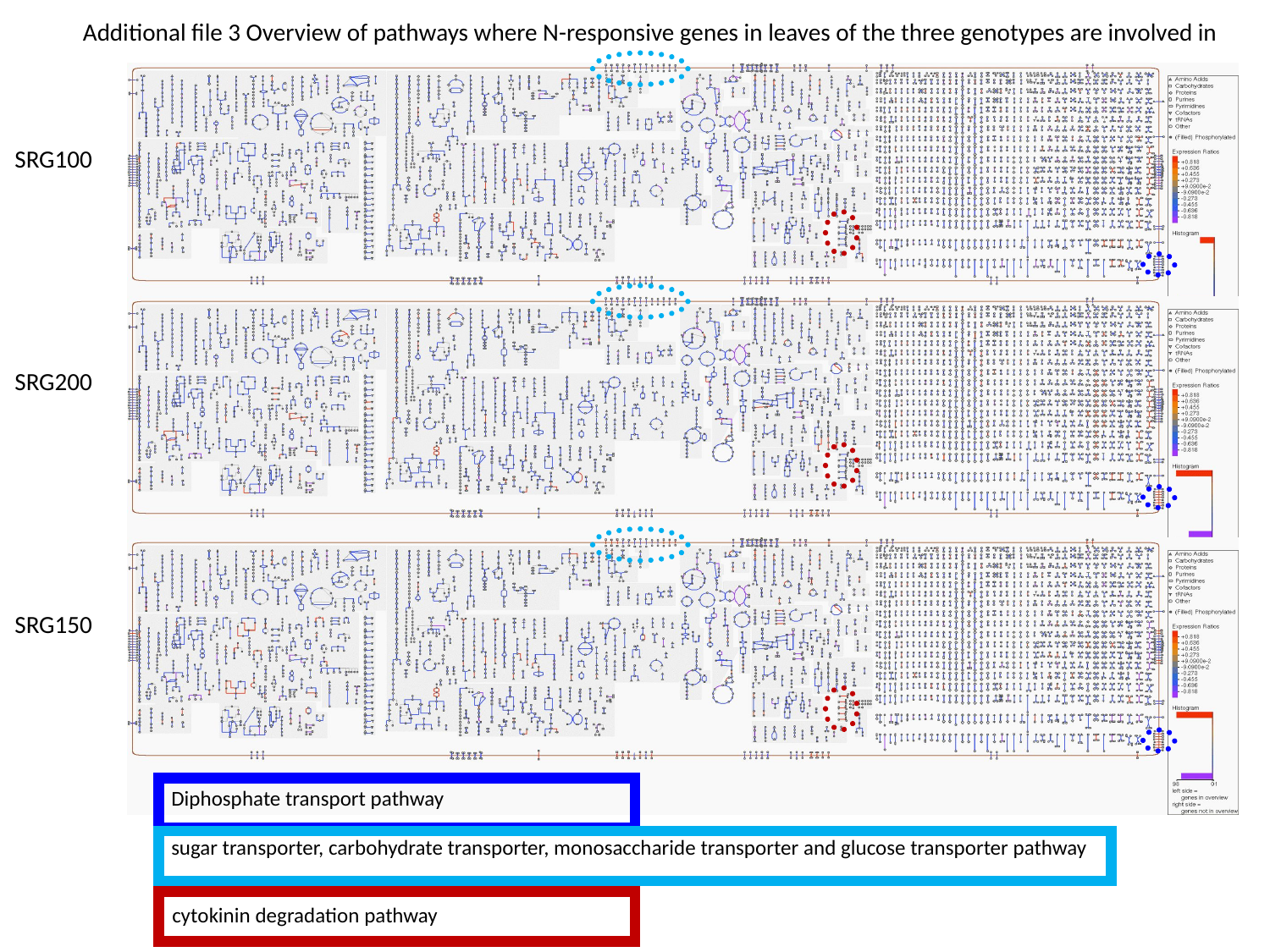

Additional file 3 Overview of pathways where N-responsive genes in leaves of the three genotypes are involved in
SRG100
SRG200
SRG150
Diphosphate transport pathway
sugar transporter, carbohydrate transporter, monosaccharide transporter and glucose transporter pathway
cytokinin degradation pathway
