# Supplementary material for: High throughput RNA sequencing of a hybrid maize and its parents shows different mechanisms responsive to nitrogen limitation
Source: BMC Genomics. 2014 Jan 28;15:77. doi: 10.1186/1471-2164-15-77 (PMC3912931; doi:10.1186/1471-2164-15-77)
Supplement: Additional file 8 — Selected significantly enriched GO terms in leaves under sufficient or limiting N conditions. [file 1471-2164-15-77-S8.doc]

| **Additional file 8. Selected significantly enriched GO terms in leaves under sufficient or limiting N conditions** | | | | | | | | | | |
| --- | --- | --- | --- | --- | --- | --- | --- | --- | --- | --- |
|  |  |  | **High N** | | | | **Low N** | | | |
| **GO Term** | **Onto** | **Description** | **1** | **2** | **3** | **4** | **5** | **6** | **7** | **8** |
| GO:0009628 | P | response to abiotic stimulus |  |  |  |  |  |  |  |  |
| GO:0016051 | P | carbohydrate biosynthetic process |  |  |  |  |  |  |  |  |
| GO:0000271 | P | polysaccharide biosynthetic process |  |  |  |  |  |  |  |  |
|  |  |  |  |  |  |  |  |  |  |  |
| GO:0044262 | P | cellular carbohydrate metabolic process |  |  |  |  |  |  |  |  |
| GO:0044271 | P | cellular nitrogen compound biosynthetic process |  |  |  |  |  |  |  |  |
|  |  |  |  |  |  |  |  |  |  |  |
| GO:0015979 | P | Photosynthesis |  |  |  |  |  |  |  |  |
| GO:0009765 | P | photosynthesis, light harvesting |  |  |  |  |  |  |  |  |
|  |  |  |  |  |  |  |  |  |  |  |
| GO:0010467 | P | gene expression |  |  |  |  |  |  |  |  |
|  |  |  |  |  |  |  |  |  |  |  |
| GO:0006796 | P | phosphate metabolic process |  |  |  |  |  |  |  |  |
| GO:0006793 | P | phosphorus metabolic process |  |  |  |  |  |  |  |  |
|  |  |  |  |  |  |  |  |  |  |  |
| GO:0019318 | P | hexose metabolic process |  |  |  |  |  |  |  |  |
| GO:0019684 | P | photosynthesis, light reaction |  |  |  |  |  |  |  |  |
| GO:0006519 | P | cellular amino acid and derivative metabolic process |  |  |  |  |  |  |  |  |
| GO:0006520 | P | cellular amino acid metabolic process |  |  |  |  |  |  |  |  |
|  |  |  |  |  |  |  |  |  |  |  |
| 1, 5 = d/a<-1 |  | expression level outside range of SRG100 |  |  |  |  |  |  |  |  |
| 2, 6 = 0> d/a >-1 |  | expression level skewed towards SRG100 |  |  |  |  |  |  |  |  |
| 3, 7 = 1>d/a>0 |  | expression level skewed towards SRG200 |  |  |  |  |  |  |  |  |
| 4, 8 = d/a>1 |  | expression level outside range of SRG200 |  |  |  |  |  |  |  |  |
|  |  |  |  |  |  |  |  |  |  |  |
| The analysis was performed using the Singular Enrichment Analysis (SEAcompare) on the AgriGO website (Du et al., 2010, http://bioinfo.cau.edu.cn/agriGO/). | | | | | | | | | | |
| This tool allowed the identification of GO terms that were significantly enriched in the lists of entities. | | | | | | | | | | |
| The false discovery rate (FDR) and the number of entities (Num) are shown where the GO term enrichment was significant. | | | | | | | | | | |
| In those cases, the cells in the table are filled with increasing shades of red as the FDR decreases. | | | | | | | | | | |
| Only some of the GO terms involved in the biological process (P) are presented here. | | | | | | | | | | |
