# Supplementary material for: High throughput RNA sequencing of a hybrid maize and its parents shows different mechanisms responsive to nitrogen limitation
Source: BMC Genomics. 2014 Jan 28;15:77. doi: 10.1186/1471-2164-15-77 (PMC3912931; doi:10.1186/1471-2164-15-77)
Supplement: Additional file 10 — Selected enriched GO terms in the hybrid expressing SRG100 or SRG200 alleles under different N conditions. [file 1471-2164-15-77-S10.doc]

| **Additional file 10. Selected enriched GO terms in the hybrid expressing SRG100 or SRG200 alleles under different N conditions** | | | | | | | | | | | | | |  |
| --- | --- | --- | --- | --- | --- | --- | --- | --- | --- | --- | --- | --- | --- | --- |
|  |  |  |  |  |  |  |  |  |  |  |  |  |  |  |
|  |  |  | **high N** | | **low N** | | **high N** | | | | **low N** | | | |
|  | | | **SRG100 alleles** | **SRG200 alleles** | **SRG100 alleles** | **SRG200 alleles** | **SRG100 alleles** | | **SRG200 alleles** | | **SRG100 alleles** | | **SRG200 alleles** | |
| **GO Term** | **Onto** | **Description** |  |  |  |  | **FDR** | **Num** | **FDR** | **Num** | **FDR** | **Num** | **FDR** | **Num** |
|  |  |  |  |  |  |  |  |  |  |  |  |  |  |  |
| **In leaves** |  |  |  |  |  |  |  |  |  |  |  |  |  |  |
| GO:0034641 | P | cellular nitrogen compound metabolic process |  |  |  |  | 0.00016 | 37 | 0.000000028 | 51 | --- | --- | 0.000085 | 28 |
| GO:0009765 | P | photosynthesis, light harvesting |  |  |  |  | 0.0073 | 8 | --- | --- | 0.000032 | 8 | --- | --- |
| GO:0019684 | P | photosynthesis, light reaction |  |  |  |  | --- | --- | --- | --- | 0.0026 | 8 | --- | --- |
| GO:0015979 | P | Photosynthesis |  |  |  |  | --- | --- | 0.014 | 18 | 0.000054 | 14 | 0.00031 | 15 |
| GO:0019725 | P | cellular homeostasis |  |  |  |  | --- | --- | 0.0000031 | 28 | --- | --- | 0.0084 | 14 |
| GO:0045454 | P | cell redox homeostasis |  |  |  |  | --- | --- | 0.0000053 | 26 | --- | --- | 0.0041 | 14 |
| GO:0019318 | P | hexose metabolic process |  |  |  |  | --- | --- | 0.0085 | 29 | --- | --- | --- | --- |
| GO:0006006 | P | glucose metabolic process |  |  |  |  | --- | --- | 0.0096 | 27 | --- | --- | --- | --- |
| **In roots** |  |  |  |  |  |  |  |  |  |  |  |  |  |  |
| GO:0034641 | P | cellular nitrogen compound metabolic process |  |  |  |  | 0.0044 | 35 | 0.000093 | 43 | --- | --- | 0.036 | 19 |
| GO:0045184 | P | establishment of protein localization |  |  |  |  | 0.0062 | 41 | 0.019 | 41 | 0.014 | 22 | --- | --- |
| GO:0015031 | P | protein transport |  |  |  |  | 0.0062 | 41 | 0.019 | 41 | 0.014 | 22 | --- | --- |
| GO:0065003 | P | macromolecular complex assembly |  |  |  |  | 0.013 | 40 | --- | --- | 0.0058 | 24 | --- | --- |
| GO:0044085 | P | cellular component biogenesis |  |  |  |  | 0.017 | 45 | --- | --- | 0.0058 | 27 | --- | --- |
| GO:0022607 | P | cellular component assembly |  |  |  |  | 0.02 | 40 | --- | --- | 0.0058 | 24 | --- | --- |
| GO:0031497 | P | chromatin assembly |  |  |  |  | 0.023 | 21 | --- | --- | 0.036 | 12 | --- | --- |
| GO:0065004 | P | protein-DNA complex assembly |  |  |  |  | 0.023 | 21 | --- | --- | 0.036 | 12 | --- | --- |
| GO:0006412 | P | Translation |  |  |  |  | --- | --- | 0.014 | 78 | 0.04 | 36 | 0.00053 | 46 |
| GO:0051246 | P | regulation of protein metabolic process |  |  |  |  | --- | --- | 0.019 | 16 | --- | --- | 0.0088 | 11 |
|  |  |  |  |  |  |  |  |  |  |  |  |  |  |  |
| The analysis was performed using the Singular Enrichment Analysis (SEAcompare) on the AgriGO website (Du et al., 2010, http://bioinfo.cau.edu.cn/agriGO/). | | | | | | | | | | | | | |  |
| This tool allowed the identification of GO terms that were significantly enriched in the lists of entities. | | | | | | | | | | | | |  |  |
| The false discovery rate (FDR) and the number of entities (Num) are shown where the GO term enrichment was significant. | | | | | | | | | | | | |  |  |
| In those cases, the cells in the table are filled with increasing shades of red as the FDR decreases. | | | | | | | | | | | | |  |  |
| Only some of the GO terms involved in the biological process (P) are presented here. | | | | | | | | | | | | |  |  |
